# To Correspondents

**Published:** 1858-01

**Authors:** 


### HOMŒOPATHY AND THE CHICAGO CITY HOSPITAL.

We see that several of our exchanges are representing this city as the only one in the country containing a public hospital under homœopathic charge. We would inform all such that our city hospital is not yet under the charge of any medical faculty. It has never been opened for the reception of patients, and we do not know when it will be.

